# Alternative ATPase domain interactions in eukaryotic Hsp70 chaperones

**DOI:** 10.3389/fmolb.2023.1155784

**Published:** 2023-03-16

**Authors:** Yassin Ben-Khoud, Chao-Sheng Chen, Maruf M. U. Ali

**Affiliations:** Department of Life Sciences, Imperial College London, London, United Kingdom

**Keywords:** Hsp70, eukaryotic chaperones, BiP, IRE1, Tim44, XIAP, HLA-DR, protein mechanisms

## Abstract

Hsp70 molecular chaperones are essential components for maintaining protein homeostasis within cells. They interact with substrate or client proteins in a well characterised fashion that is regulated by ATP and supported by co-chaperones. In eukaryotes there is a vast array of Hsp70 isoforms that may facilitate adaption to a particular cellular compartment and distinct biological role. Emerging data indicate a novel type of interaction between Hsp70 and client protein that does not fit with the classical Hsp70 ATP regulated substrate mechanism. In this review, we highlight Hsp70 ATPase domain interactions with binding partners from various biological systems that we refer to as **H**sp70 **A**TPase **a**lternative **b**inding proteins or HAAB proteins. We identify common mechanistic features that may define how Hsp70 operates when associating with proteins in this alternative HAAB mode of action.

## 1 Introduction

Hsp70 chaperones are essential components of processes that maintain the quality of proteins within the cell. They are involved in extensive range of functions that include the folding of newly synthesised proteins, the transport of nascent proteins to different cellular compartments such as the mitochondria and ER, act to prevent protein aggregation, and enhance refolding of misfolded proteins in both stressed and non-stressed cellular conditions. Due to their central role in protein maintenance, any aberrations in their function will have a hugely deleterious effect on cell fitness that may lead to diseases such as Alzheimer’s and aging (Hartl et al., 2011; Hipp et al., 2019; Rosenzweig et al., 2019).

There are multiple isoforms of Hsp70 chaperones within the eukaryotic cell. They are assisted in their action by an even greater number of cochaperones that enable Hsp70 complexes to interact with a variety of substrate proteins. These substrates are typically unfolded, misfolded or denatured proteins that expose a short extended hydrophobic motif that Hsp70 associates with and are termed client proteins. Many years of careful research has pieced together how Hsp70 chaperone complexes interact with client proteins in an ATP dependent manner with the bulk of the early research focused on the bacterial system (Hartl et al., 2011; Rosenzweig et al., 2019).

However, owing to the vast diversity found within eukaryotes, certain Hsp70 chaperones can adapt to a distinct cellular location to act in a particular way. This manifests in Hsp70 interactions that do not fit with the classical client and ATP regulated binding mode and represent a specialised protein interaction that may serve a distinct biological role. The molecular details of such non-canonical interactions are yet to be clearly understood (Larburu et al., 2020; Johnson and Gestwicki, 2022). In this review, we focus on interactions mediated *via* the ATPase domain of Hsp70 with emphasis on recently characterised associations involving the ER Hsp70 chaperone, BiP, and discuss other adapted eukaryotic Hsp70 systems.

## 2 Classical Hsp70 client interactions

Hsp70 chaperones undergo coordinated movements that facilitate the binding and release of client proteins. Remarkably, they have a well conserved molecular structure across all species and isoforms that consist of a nucleotide binding domain (NBD) also referred to as the ATPase domain, a substrate binding domain (SBD) and a linker that connects the two domains (Flaherty et al., 1990; Kityk et al., 2012). The NBD is made up of four subdomains that form together to create a cleft that mediates the binding of ATP. The binding and hydrolysis of ATP facilitates subtle movements within the active site cleft that propagates throughout the NBD (Buchberger et al., 1995; Mayer, 2018; Mayer, 2021). Hydrolysis of ATP by Hsp70 is slow with typically one ATP turnover in 20–30 min (Zuiderweg et al., 2017; Mayer, 2018; Mayer and Gierasch, 2019). The SBD directly binds to client proteins by engaging a short hydrophobic motif (Rosenzweig et al., 2019). The SBD is further divided into two subdomains, SBDα and SBDβ. The SBDβ is made up of an eight stranded β-sandwich fold that contains a hydrophobic pocket that constitutes the polypeptide binding pocket. The SBDα is a helical structure that acts as a lid enclosing the substrate within the SBD (Zhu et al., 1996; Bertelsen et al., 1999).

**FIGURE 1:**
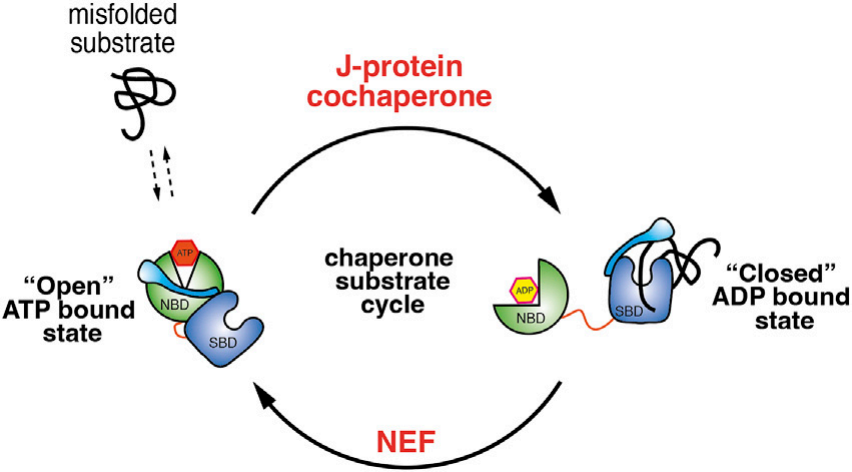
Illustration depicting the Hsp70 ATPase cycle. When Hsp70 is bound with ATP it adopts a conformation that positions the NBD and SBD in proximity. This facilitates the movement of lid, exposing the substrate binding pocket, which enables fast substrate binding and release and works together with J-domain co-chaperones that recruits misfolded protein to Hsp70. The association of J-domain co-chaperone stimulates ATP hydrolysis and transition to ADP bound conformation in which the lid now closes over the SBD trapping the substrate. NEF mediate the exchange of ADP for ATP and allows transition back to the “open” ATP bound Hsp70 state.

The binding and release of client proteins from the SBD are allosterically coupled to binding and hydrolysis of ATP. In the ATP bound state, Hsp70 has a low affinity for its substrate with a high K_on_ and K_off_ rate. Upon hydrolysis of ATP to the ADP bound state, Hsp70 undergoes structural rearrangement that places the SBDα lid over the substrate leading to an increase in affinity with a low K_on_ and K_off_ rate (Mayer and Gierasch, 2019; Rosenzweig et al., 2019; Mayer, 2021). There are two sets of co-chaperones that facilitate the transition between the ATP and ADP bound states. First, J-domain proteins that stimulate the hydrolysis of ATP by Hsp70 and stabilise the substrate association (Laufen et al., 1999; Kampinga and Craig, 2010). Second, nucleotide exchange factors or NEF that mediate the exchange of ADP to ATP thus enabling the release of bound substrate from SBD (Laufen et al., 1999) (Figure 1). Therefore, it is the concerted action of co-chaperones and the binding of substrates that increases the rate Hsp70 ATP hydrolysis.

## 3 Hsp70 NBD interactions in ER stress signalling

The unfolded protein response (UPR) is a cell signalling system that detects the presence of misfolded proteins within the ER and coordinates a cellular response that aims to restore protein homeostasis. These responses include an increase in transcription of UPR target genes such as chaperones that will assist in clearing misfolded proteins 2) brief attenuation of protein translocation by inhibiting the ribosome 3) increase in ER associated degradation 4) and an enlargement of the ER that facilitates a greater surface area (Wang and Kaufman, 2012; Adams et al., 2019; Hetz et al., 2020).

There are three receptor proteins that are essential for initiating the UPR response: IRE1, PERK, and ATF6. All three proteins straddle across the ER membrane presenting a luminal domain (LD) that is involved in detecting misfolded proteins; a single pass transmembrane region; and a cytosolic portion that is responsible for propagating the signal. The LD of IRE1 and PERK share a high degree of structural similarity suggesting that they may operate the same mechanism for detecting misfolded proteins (Credle et al., 2005; Zhou et al., 2006; Carrara et al., 2015a). The UPR receptor proteins have been shown to interact with the ER Hsp70 chaperone BiP and that ER stress reduces the association (Kozutsumi et al., 1988; Bertolotti et al., 2000; Oikawa et al., 2009; Hetz et al., 2020).

The mechanism of UPR induction is still debated [for UPR activation reviews see Adams et al. (2019), Preissler and Ron (2018), and Karagoz et al. (2019)]. There are two BiP dependent models that differ in the way they associate with BiP. In this review, we focus on the allosteric model that suggests IRE1 interacts with BiP primarily through its NBD domain (Carrara et al., 2015b; Kopp et al., 2018). This interaction was shown to be independent of nucleotides, with the binding affinity between the LD and BiP unaffected by the presence of ATP, ADP and AMP-PNP. The addition of C_H_1—an antibody fragment that is inherently misfolded in the absence of its cognate binding partner C_L_–caused the complex to breakdown. C_H_1 was shown to bind to BiP within the canonical SBD polypeptide pocket as a substrate (Marcinowski et al., 2011; Carrara et al., 2015b). The binding of substrate to the BiP SBD caused the dissociation of BiP NBD from IRE1 LD *via* a likely allosteric change that lowered the affinity between the proteins. To further demonstrate differences in way BiP interacts with misfolded substrate proteins by means of the SBD and non-canonical binding *via* the NBD, IRE1 LD was heat denatured and its association to BiP (BiP^WT^) and a BiP mutant (BiP^V461F^) was assessed. The V461F mutation is located within the SBD and prevents engagement of misfolded substrates to BiP (Petrova et al., 2008). Robust binding was observed between native IRE1 LD to both BiP^WT^ and BiP^V461^ (K_d_ = 0.54 μm and K_d_ = 0.7 μm). However, heat denatured IRE1 LD bound to BiP^WT^ with a reduced affinity (K_d_ = 2.9 μm) and was unable to bind to BiP^V461^, suggesting that native and misfolded IRE1 LD interacts with BiP in a different way. Together, the data indicates that IRE1 LD interacts *via* the ATPase domain of BiP and its association is distinct from the canonical substrate protein binding mediated by the SBD (Carrara et al., 2015b; Kopp et al., 2018; Kopp et al., 2019).

Although nucleotides do not affect IRE1 LD association to BiP, they do play a critical role. In experiments where BiP and IRE1 LD proteins were N-terminally fused with yellow fluorescence protein (YFP) and cyan fluorescence protein (CFP), their association generated a FRET signal (Kopp et al., 2018). Nucleotides did not alter the FRET signal, again suggesting that they do not impact IRE1 LD–BiP association (Kopp et al., 2019). The addition of C_H_1 caused the loss of FRET signal by inhibiting binding between BiP and IRE1 LD. Furthermore, when measuring the loss of FRET on addition of increasing concentrations of C_H_1 in the presence and absence of nucleotides, there was a 21-fold decrease in the concentration of C_H_1 required to cause full dissociation of IRE1 LD-BiP complex in the presence of ATP when compared to ADP and APO states (Kopp et al., 2019). Thus, suggesting that ATP primes BiP-IRE1 complex to detect misfolded protein leading to dissociation from IRE1.

The binding of IRE1 to the NBD switches BiP from acting as a chaperone to operating as a stress sensor. BiP has a low-level ATPase activity that is stimulated by its co-chaperones such as the J-domain protein ERdj3 and its NEF SiL1. However, the addition of IRE1 LD resulted in the loss of stimulation suggesting competitive binding between IRE1 and BiP co-chaperones (Kopp et al., 2019). An NBD mutation based on the BiP-Sil1 co-crystal structure (Yan et al., 2011) was shown to affect binding of both co-chaperones and IRE1 LD, thus providing further evidence of binding to NBD. By preventing the binding of J-protein and NEF, IRE1 inhibits BiP functioning as a chaperone, whilst ATP would prime BiP to engage misfolded protein (Kopp et al., 2019). The binding of misfolded protein is coupled to dissociation from IRE1, which would subsequently enable IRE1 to oligomerise and activate UPR (Bertolotti et al., 2000; Oikawa et al., 2009; Carrara et al., 2015b). Thus, the biological role of the specialised non-canonical interaction between BiP NBD and IRE1 would be to enable BiP to act as an sensor in ER stress signalling (Adams et al., 2019).

## 4 Hsp70 NBD interaction in the PAM motor complex

The passage of nascent polypeptide into mitochondrial matrix occurs post translationally. The newly synthesized polypeptide is engaged by chaperones after its emergence from the ribosome in the cytosol where it is kept in an extended conformation until it associates with the mitochondrial outer membrane complex termed the TOM complex. After passing through the TOM complex, it encounters the inner membrane translocation complex known as TIM23. Here, the negative potential across the inner membrane along with the action of a dedicated motor complex call the PAM complex help to drive through the positively charged polypeptide into mitochondrial matrix where it can attain its native structure (Craig, 2018; Pfanner et al., 2019).

The PAM complex is composed of five essential subunits in yeast. Three of the subunits are a part of the Hsp70 chaperone system consisting of the mitochondrial Hsp70 (mtHsp70), along with its dedicated NEF and J-protein co-chaperones. The other two essential components are Tim16 and Tim44. They both act as scaffold proteins that make multiple contacts with other PAM proteins to ensure the structural integrity of the motor complex. Tim44 is made up of two domains, N and C terminal domains, that are roughly equivalent in size (Craig, 2018; Pfanner et al., 2019).

Tim44 primarily interacts with mtHsp70 *via* its NBD domain (Krimmer et al., 2000; Strub et al., 2002). This association maybe supplemented by interactions with part of the SDB, but not as a chaperone substrate, as the binding of substrate peptide did not affect the interaction of mtHsp70 to Tim44 (D’Silva et al., 2004).

Interestingly, *in vitro* analysis indicated that the interaction was independent of nucleotides. However, upon addition of peptide substrate, the mtHsp70-Tim44 association was destabilised in the presence of ATP but remained stable with ADP indicating that ATP may prime the complex towards binding substrate peptide (D'Silva et al., 2004; Liu et al., 2003). The association between mtHsp70 NBD and Tim44 enables Hsp70 to be optimally positioned to engage the nascent polypeptide *via* its SBD as it emerges through the pore. Upon binding the substrate, Hsp70 would release from Tim44 and the confines of the membrane to facilitate entropic pulling of the nascent chain out of the pore and into the mitochondrial matrix (Craig, 2018). Thus, mtHsp70-Tim44 interaction plays a role in protein export across membrane and into the mitochondrial matrix.

## 5 Hsp70 NBD interactions in apoptosis signalling

The inhibitor of apoptosis proteins (IAP) were initially identified as baculovirus proteins that inhibited the process of apoptosis and cell death within the insect cell host upon baculovirus infection (Crook et al., 1993). There are several members of the IAP family including c-IAP1, c-IAP2 and X-linked IAP (XIAP). They are characterised by possessing at least one baculoviral IAP repeat domain (BIR) along with a C-terminal ring finger motif (Hinds et al., 1999) within their protein sequence. The BIR domain mediate protein-protein interactions most notably with caspases, an association that ultimately leads to inhibition of apoptotic signalling. XIAP was identified as a client substrate protein of Hsp70 based on experiments where small molecule inhibitors of Hsp70 activity led to the degradation of XIAP (Cesa et al., 2018) and by point mutations located within the BIR domain showing a weakened affinity towards Hsp70 (Cesa et al., 2018). Interestingly, truncated XIAP that contained only the BIR domains, BIR2 and BIR3 (XIAP_120-356_), did not exhibit binding to Hsp70 SBD when analysed by NMR chemical shift perturbations (Cesa et al., 2018). Competitive fluorescence polarisation experiments showed that addition of a substrate peptide did not inhibit binding of XIAP_120-356_ to Hsp70, which again demonstrated that binding between XIAP_120-356_ and Hsp70 was not mediated *via* the canonical SBD domain (Cesa et al., 2018). Furthermore, the interaction between XIAP_120-356_ and Hsp70 was not strongly affected by the presence of nucleotides. The rationale put forth was that the binding sequence contained within the BIR domain may not be in an extended and partially unfolded state to which Hsp70 could bind as a substrate, but that binding occurs in an alternative manner. Evidence to support this idea comes from the observation that Hsp70 NBD (lacking the SBD) was sufficient to disrupt binding between Hsp70 and the XIAP_120-356_ and as a result of disturbing the non-canonical interaction led to degradation of XIAP (Cesa et al., 2018; Johnson and Gestwicki, 2022). Thus, from the data XIAP binds to the NBD of Hsp70, but it is unclear as to what the biological role of this mode of binding is.

## 6 Hsp70 NBD interactions in antigen presentation

HLA-DR is an MHC-II receptor protein involved in antigen presentation and T-cell stimulation (Thorsby, 1984). Peptides derived from a hypervariable region present within HLA-DR molecules are known to associate with Hsp70 possibly to mediate transfer of peptide to MHC-II protein although this is not clearly understood (Auger et al., 1996). Mutations in the SBD resulted in reduced binding to HLA-DR peptides indicating that binding occurs *via* the canonical Hsp70 SBD. Similarly, full-length HLA-DR was observed to be able to bind to Hsp70 (Haug et al., 2007). In binding competition experiments the addition of peptide did not affect the association of labelled HLA-DR to Hsp70, however, there was a loss of binding when unlabelled HLA-DR was titrated in, suggesting that association may not occur in a similar manner to peptide binding *via* Hsp70 SBD (Haug et al., 2007). Moreover, the binding of Hsp70 to peptides was abrogated by addition of ATP, but the association with HLA-DR was unaffected, indicating a different binding mode between full length HLA-DR and short peptides derived from the HLA-DR protein to Hsp70 (Haug et al., 2007). In addition, quantitative binding experiments demonstrated that constructs lacking part of the Hsp70 SBD inhibited binding to peptides but did not impact binding to HLA-DR, and that the isolated Hsp70 NBD displayed similar binding affinity to full length Hsp70 (Rohrer et al., 2014). The experimental evidence suggests that binding between HLA-DR and Hsp70 occurs *via* the NBD; however, it is less clear what the biological significance of this interaction is, with the suggestion that it may enable the formation of a Hsp70-peptide-HLA-DR ternary complex that would facilitate the transfer of peptide to the binding groove within the HLA-DR (Haug et al., 2007).

## 7 Emerging NBD interactions

Recent data indicates many new interacting proteins that associate with Hsp70 *via* its NBD. A mass spectrometry analysis of cross-linked Hsp70 from yeast identified numerous proteins from diverse systems such as cell cycle control to chromatin organisation that interacted with NBD, and that some of the interactions were influenced by post translational modification (PTM) (Nitika et al., 2022). The biological significance of the interactions are yet to be fully explored. Thus, emerging data suggest many new NBD interactions and that some maybe regulated by PTM in a process termed the “chaperone code” (Nitika et al., 2020).

## 8 Common features of alternative ATPase domain non-canonical association

**FIGURE 2:**
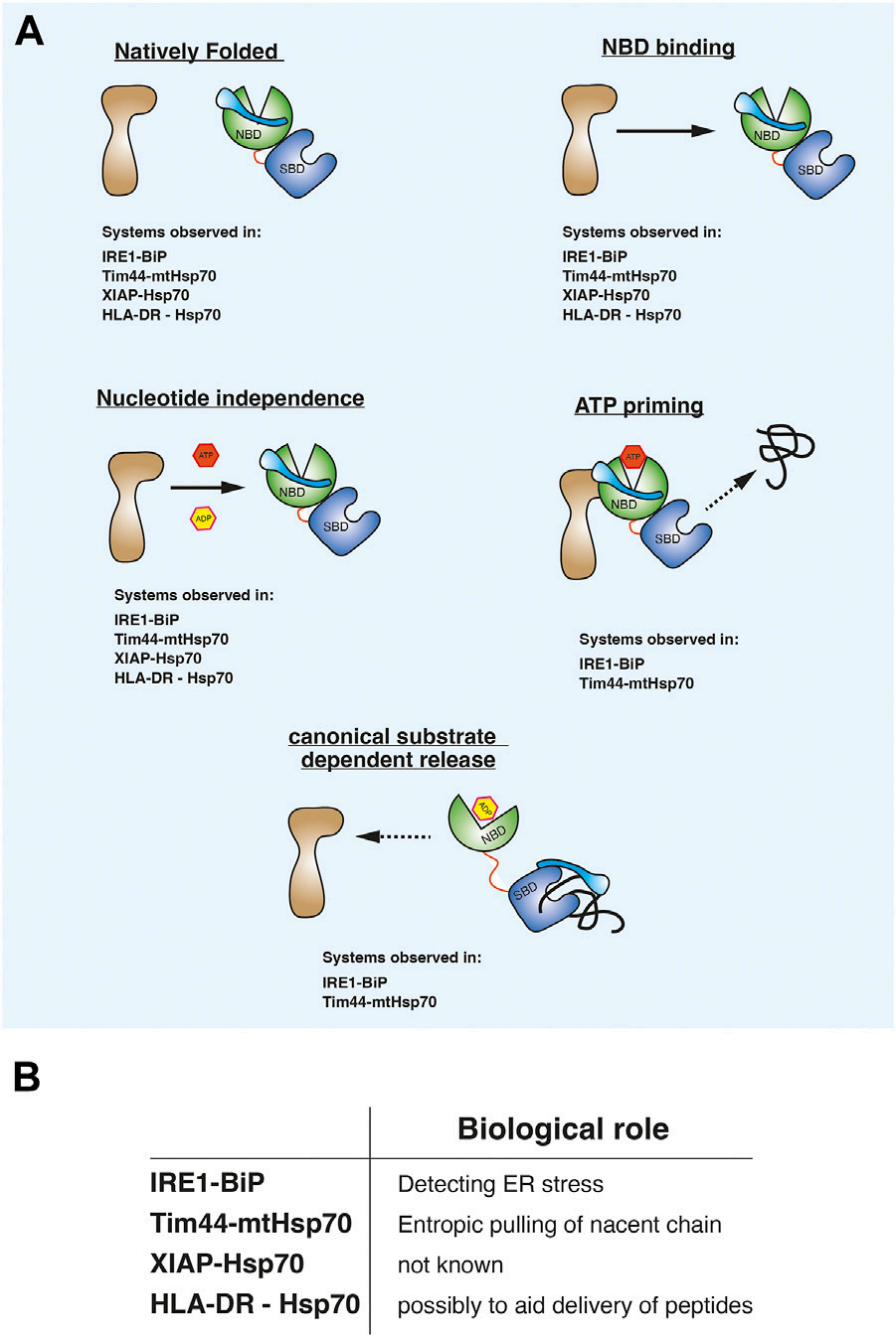
**(A)** diagram showing common mechanistic features that underlie how Hsp70 operates when associating with proteins that do not fit the classical substrate client mode of action. We refer to them as Hsp70 ATPase alternative binding protein or HAAB proteins and Hsp70 HAAB mechanism of action. **(B)** Possible biological roles for Hsp70 in alternative binding mode.

Evaluating the observations from the different biological systems suggests that there may be a consistent pattern in the way Hsp70 associates in this alternative binding mode. First, the non-canonical binding partner is in a native folded state and not misfolded, in an extended conformation, or in a non-native intermediate condition. Second, the non-canonical interaction is mediated predominately *via* the NBD domain. Third, the association between Hsp70 NBD and binding partner is unaffected by nucleotides. Fourth, ATP may prime the Hsp70-complex to interact with substrate. Fifth, misfolded substrate proteins or peptides bind to the canonical SBD which causes the release of the NBD mediated non-canonical interaction (Figure 2A). The last two points are observed with the ER and mitochondrial Hsp70 systems for which there is a biological rationale for providing an alternative binding mode. It is yet to be elucidated what the role of this binding is for the XIAP and HLA-DR, although for HLA-DR it has been suggested to possibly assist in the transfer of peptides during T-cell stimulation (Figure 2B). The common features comprise a distinct mechanism underpinning the ATPase domain interaction that differs from the classical substrate chaperone regulation and therefore referring to this type of interacting protein as a client protein can be unclear. Furthermore, these binding partners are not co-chaperones that accelerate Hsp70 ATPase activity *via* interactions with the NBD. To aid clarity, we refer to them as **H**sp70 **A**TPase **a**lternative **b**inding proteins or HAAB proteins. HAAB consist of a group of Hsp70 binding proteins that enable Hsp70 to operate with the aforementioned mechanistic characteristics that differs from its canonical interaction with client proteins. Thus, different adapted Hsp70 systems may have common features that underlie how ATPase domain association enable the Hsp70 chaperones to operate in an alternative HAAB mechanism.

## 9 Summary

In this review, we describe a Hsp70 non-canonical binding mode that has only been observed in Eukaryotes. This is most likely as a result of adaptions forged out of the diverse cellular environments that can enable alternative biological functions. Our understanding of such interactions and the role that they serve are still far from complete.

A better understanding of the precise binding site that mediates the non-canonical association is required and how this alternative binding mode impacts known interactions with Hsp70 co-chaperone that associate within NBD and what role PTM’s play in regulating this interaction is needed. Furthermore, a clearer understanding on what the biological purpose of such associations are and what bearing this has on Hsp70 chaperone system overall. By highlighting common features between these diverse Hsp70 interacting systems that we refer to as HAAB proteins, we hope it acts as a springboard to continue to gain understanding; particularly, as it could be critical for healthy protein homeostasis in human cells and could hold promising therapeutic opportunities in disease.
